# Sex testing in women's sport: historical harms, contemporary risks, and World Athletics' 2025 policy shift

**DOI:** 10.3389/fspor.2025.1723127

**Published:** 2026-02-10

**Authors:** Silvia Camporesi, Marcus Mazzucco, Maria José Martínez Patiño, Jonathan Ospina-Betancurt, Sarah Teetzel

**Affiliations:** 1 Interdisciplinary Centre for Ethics, Regulation and Integrity in Sport, Centre for Biomedical Ethics and Law, KU Leuven University, Leuven, Belgium; 2 Faculty of Kinesiology and Physical Education, University of Toronto, Toronto, ON, Canada; 3 Faculty of Education and Sports Sciences, University of Vigo, Pontevedra, Spain; 4 Departamento Didáctica de la Expresión Musical, Plástica y Corporal, Facultad de Educación, Universidad de Valladolid (UVA), Valladolid, Spain; 5 Faculty of Kinesiology and Recreation Management, University of Manitoba, Winnipeg, MB, Canada

**Keywords:** athlete rights, female sport, sex testing, SRY, variation of sex characteristics, women's sport

## Abstract

The history of systemic, mandatory sex testing of all women athletes in sport is now well documented, with hundreds of articles, entire books, and doctoral dissertations analyzing the scientific, legal, and ethical aspects of sports governing bodies' determination to verify that competitors in the women's event were women and that men did not sneak into women's sports. A 30 July 2025 press release from World Athletics (WA), announcing changes to its eligibility rules for competing in female events is momentous for three reasons. First, in referring to women's events specifically and exclusively as female events it signals that the organization prioritizes biological sex, not gender, in its eligibility considerations. Second, it merges regulations for women with variations of sex characteristics ^1^ and transgender women into one set of regulations, ignoring the differences between these two groups of women athletes. Finally, it marks the return of systemic sex testing for all elite women athletes competing in track and field, a practice that ended in the 1990s for good reasons, given the substantial legal, ethical, and cultural objections voiced by a myriad of medical and scientific bodies, as well as by athletes who bravely shared their experiences. In critically examining WA's press release outlining its decision to reinstate systemic sex testing through mandatory screening of the Sex-determining Region Y (SRY) gene for all high-performance women track and field competitors, we demonstrate how these regulations reproduce patterns of harm condemned by experts in the fields of medicine, ethics, and human rights. We conclude that WA's July 2025 decision fails to address the cogent arguments that led to the cessation of systemic sex testing in 1999, contravenes human rights laws, and anachronistically reintroduces harmful practices that were abandoned for good reasons.

## Introduction

“When I came back to Spain a doctor, member of the Spanish expedition, told a journalist [I had failed the sex testing]. The journalist published the event in newspapers and magazines, T.V. etc. And as a result of that, everybody knew about it. My federation [did] not support me, and they [expelled] me from the residence sport (the place where I lived). I left the University and so I had to work because I had no money to live. I have lost my friends, and my boyfriend left me when the event came to light; Twelve years of hard work for nothing. I thought I was going to die. I lost my licence and my performances. It looked as if I were a man instead of a woman…I understand it is very difficult to obtain a modification of [eligibility] of [WA]^2^ but I think, this is not impossible consider special cases. I hope my sacrifice will not be left in oblivion. While I live I'll go on fighting. But I am alone. I need your help. I need your support. I know deeply your works based on medical research about the subject in Cell, Jama, Lancet. I have sent a letter to Mr. Samaranch and I Have the acknowledgment of the King of Spain. I would like that this case were known by specialist in Genetics and sports Medicine. The doctors have given me the impulse to continue fighting, and they have [said] that my [disqualification] is not Fair; the results confirm: I am really a woman, even so my federation not let me participate in competitions with woman. Please, sir, I need your help… I wonder what are my possibility and if it is possible, before I die, to compete [again]… I will go on fighting and I Hope to find someone in the world that will help me… You are my best and most important help for me. I have nothing to hide. I have not deceived anybody; and I felt ashamed and humiliated… I have lost everything and I need your help.” (Letter to Dr. Albert de la Chapelle from María José Martínez Patiño, 25 March 1988.^3^ See Supplementary file 1)

Nearly 40 years after Spanish hurdler María José Martínez Patiño contacted Dr. Albert de la Chapelle with a plea for help, her anguish is still difficult to read. The raw emotions of a then 24-year-old young woman, going out on a limb to seek assistance from a renowned geneticist from University of Helsinki's Department of Medical Genetics, are evident in the letter she sent. Five years prior to the time of writing, as an emerging 19-year-old athlete selected to represent Spain at the 1983 World Championships in Helsinki, Finland, Martínez Patiño obtained her “femininity card” after passing WA's required sex screening process. Her results in Helsinki were modest, where she did not challenge the top hurdlers and sprinters for gold medals. Her times of 13.78 in her qualifying heat of the 100 m hurdles and 13.80 in the quarterfinal placed her 6th of 7 athletes and 7th of 8, respectively, nearly half of a second behind the cut-off time to advance to the semi-finals, and 1.5 s behind the eventual winner. While delivering respectable results, the teen athlete was not a medal contender or threat.

After overcoming serious family and health issues, Martínez Patiño was selected to represent Spain at the 1985 University Games in Japan. At these games, she forgot her sex test documentation issued in Helsinki and was required to undergo a new test. When that test detected chromosomal patterns other than XX, WA's decision to ban her from competing in future women's events was leaked to a reporter and publicized. In her plea to the influential geneticist above, Martínez Patiño outlined some of the devastating implications, beyond athletic ineligibility, that stemmed from WA's decision.

Dr. Albert de la Chapelle's response was helpful, informative, and provided hope for the young Spanish athlete whose life had been altered permanently and negatively by WA's decision. He offered not only his scientific expertise as a leading genetics researcher, but also his compassionate opinion on the ethics and appropriateness of using the sex chromatin test for the purpose of deciding sports eligibility:

“Your case and those of many, many others is a tragic illustration of the inadequacy of the sex chromatin screening procedure. I and many other scientists have for many years tried to convince the sports organizations to abandon the sex chromatin screening so that further mistakes of this type can be avoided. Unfortunately, the sports organizations, notably the IOC and the [WA], have not yet decided to abandon the sex chromatin screening. They have refused to do so even after they have come under severe pressure from scientific organizations, which have passed resolutions asking them to reconsider their procedures in order to avoid further human suffering” (Letter to Maria José Martínez Patiño from Albert de la Chapelle, M.D., 8 March 8 1988.^4^ See Supplementary file 2)

As de la Chapelle wrote in his response to Martínez Patiño in 1988, efforts by the International Olympic Committee (IOC) and WA to regulate eligibility in women's sports events using sex testing methods were considered by the scientific and medical communities to be “tragic” and direct causes of “human suffering”. Martínez Patiño's second sex chromatin test identified her karyotype as 46, XY, which led to her disqualification from competition without recognizing her condition as woman with androgen insensitivity syndrome. The impact of being told you were no longer considered a woman eligible to compete in women's sports was traumatic and life altering (1).

WA's regulations in force at the time required women to undergo a form of sex screening, known as the Barr body test, to establish that they had two X chromosomes. As noted by Krieger and colleagues (2), at the time, the “Barr body test was a new innovation that could identify an individual's chromosomes. Yet, medical practitioners, including Murray Barr himself, who invented the test, cautioned against using it as the sole determinant of sex” (p. 9–10). A similar argument, we will see, has been recently raised by Andrew Sinclair in relation to the reintroduction of genetic tests based on the presence of the Sex-determining Region Y (SRY) gene (3). Two decades after Martínez Patiño's experiences highlighted the inadequacies of using chromosome testing, and the violation of privacy that arises from the dissemination of test results, Caster Semenya was thrust into a similar spotlight at the 2009 World Athletics Championships in Berlin when media critics, sports officials, and some of her competitors questioned her right to compete in women's track and field events.

Semenya was 18 years old when she ran a gold medal winning 800 m race in an unexpectedly fast time, beating the Kenyan runner Janeth Jepkosgei by a margin of 2.45 s, and finishing the race in 1:55.45. It was her first professional competition outside of her home country, South Africa. After completing the race, Semenya was informed that she too was not a woman, or not woman enough to compete in women's sports events (4). As Semenya has put it: “They did not see me as an 18-year-old woman. They did not see me as a young girl from the bush who was the best in the world. They did not see me as human at all. They saw me as science. They wanted to test my body” (5).

The WA moved quickly to introduce regulations to limit women's functional testosterone levels, resulting in a series of regulatory policies setting endogenous testosterone thresholds, and requiring athletes whose bodies triggered suspicions to undergo individual assessments of their sex. Referred first as Hyperandrogenism Regulations (6) and eventually as Eligibility Regulations for the Female Classification [Athletes with Differences of Sex Development (DSD)] (7), WA's implementation of these regulations has kept the Court of Arbitration of Sport (CAS) busy for the past 15 years sorting through challenges brought forward regarding both the integrity of the evidence used to ground the regulations, as well as the legality of enforcing the regulations in international sport (8–10). WA introduced the Hyperandrogenism Regulations in 2011 not as – purportedly at least – a way of policing sex, but as a way of ensuring a level playing field within the women's category. In the Hyperandrogenism Regulations, there was no mention that women with elevated endogenous levels of testosterone were not considered biological females. This type of language has been introduced more recently, notably in the arguments advanced by WA in the Semenya and ASA v. IAAF case at CAS in 2019, and then in WA's new Eligibility Rule 3.5 (Male and Female Categories) and the accompanying Regulations for the Implementation of Eligibility Rule 3.5 that took effect on 1 September 2025 (9, 11–13).

Dutee Chand, an Indian sprinter competing in the 100 and 200 m, received similar news to Martínez Patiño and Semenya in 2014 when she was informed that sex screening conducted under WA's Hyperandrogenism Regulations rendered her ineligible to compete in women's events. Just days ahead of the 2014 Commonwealth Games in Glasgow, Scotland, Chand was excluded from participation and required to undergo androgen suppressive treatment to lower her testosterone levels below 10 nmol/L to be eligible to compete in women's events. Chand refused to comply with the WA's regulations and challenged them at CAS, where hearings were held in March 2015 at the CAS's headquarters in Lausanne, Switzerland. On 24 July 2015, the CAS panel issued its Interim Award suspending the Hyperandrogenism Regulations for two years on the basis that WA had not discharged its onus of establishing that the regulations were necessary and proportionate to pursue the legitimate objective of ensuring fairness in the women's competition category. More specifically, the CAS panel found that WA had “not provided sufficient scientific evidence about the quantitative relationship between enhanced testosterone levels and improved athletic performance in hyperandrogenic athletes” and that in the absence of such evidence the CAS panel was “unable to conclude that that hyperandrogenic female athletes may enjoy such a significant performance advantage that it is necessary to exclude them from competing in the female category” (8, para. 547). In line with this, independent performance studies likewise found no measurable connection between endogenous testosterone and elite women's track outcomes, including event-by-event comparisons during the period in which the Hyperandrogenism Regulations were in force from May 1, 2011 to July 27, 2015 (14, 15).

The suspension allowed the participation of Dutee Chand, as well as all women athletes with, at that time, testosterone levels above 10 nmol/L. However, a closer look at the CAS panel's decision gave reasons to worry (16). While the suspension of the regulations was obviously regarded as “good news” in the short term for Chand, it was concerning that the proviso for the suspension of the regulations effectively encouraged WA to proceed down this path of regulating eligibility of the female classification as long as they had additional evidence (17). The panel explicitly stated that WA's assumption (that increased testosterone confers an advantage) *“*may well be proved valid” (8, paragraph 534) but added that sufficient evidence had not yet been provided to show evidence of correlation. On that basis, the CAS panel ruled that the “onus of proof remains” on WA (8, paragraph 535).

WA was given 2 years (later, extended by 3 months) to produce additional evidence for the correlation between endogenous testosterone and performance advantage. However, in the end, WA decided to abandon the suspended Hyperandrogenism Regulations and replace them with the Eligibility Regulations for the Female Classification (Athletes with DSD), published on 23 April 2018 and effective 1 November 2018 (7). The 2018 regulations applied only to a subset of events (from the 400 m to the mile) and did not include the 200 m event in which Chand was competing. This we argue is an early example of WA moving the goalposts (a point we revisit later in the paper). This move achieved three aims. First, from a legal perspective, WA's decision made the CAS panel's decision moot as the suspended regulations were no longer in effect, and any challenge to the new 2018 regulations would require a new arbitration proceeding before CAS (which eventually occurred with Semenya's case). Second, this 2018 change allowed Chand to compete (although only temporarily, until the Eligibility Regulations for the Female Classification (Athletes with DSD) were revised in 2023 to apply to all events). And, third, the change allowed WA to avoid public scrutiny and avoid legal accountability for the Hyperandrogenism Regulations.

WA repeated this strategy in 2025 when it announced that it was replacing the 2023 Regulations with new sex testing regulations that would require women athletes to be screened for the SRY gene. This policy shift followed two key events. The first was an attempt by Maximila Imali, a Kenyan sprinter, to challenge the 2023 regulations at CAS (18). The second was Semenya's partial victory at the European Court of Human Rights, which opened the door for her to return to the Swiss Federal Tribunal and have them again review CAS's decision regarding the lawfulness of the 2018 regulations (19). It is possible that, when faced with the risk that the Swiss Federal Tribunal could opine on the proportionality of the 2018 regulations (which would have had implications for the more restrictive 2023 regulations), that WA decided to largely abandon its policy of using testosterone limits to determine eligibility and return to genetic testing. As a result, while Semenya's challenge to the 2018 regulations “reached the highest possible court with a highly successful outcome” (20), WA's actions make it necessary for a new legal proceeding to be commenced to challenge the 2025 regulations by an athlete who is still competing in athletics, as opposed to Semenya who has retired from the sport.

The history of systemic, mandatory sex testing of all women athletes in sport is now well documented, with hundreds of articles, entire books, and doctoral dissertations analyzing the scientific, legal, and ethical aspects of sports officials' determination to verify that women athletes were women and that men did not sneak into women's sports [e.g., (21–25)]. WA's 30 July 2025, press release announcing changes to its eligibility rules for competing in women's events was momentous for three reasons. First, in referring to women's events specifically and exclusively as female events it signals that the organization prioritizes biological sex, not gender, in its eligibility considerations. In doing so, it simplifies the complex biology of human sex to only one of its characteristics, reinforcing mistakes that were acknowledged and corrected by the IOC at the end of the last century, as we will discuss in depth below. Second, it merges regulations for women with sex variations and transgender (trans) women into one set of regulations, ignoring the differences between these two groups of women athletes. And third, it marks the return of systemic sex testing for all women athletes for all World Ranking Competitions, a practice that ended in the 1990s for good reasons, given the substantial legal, ethical, and cultural objections voiced by a myriad of national and international medical and scientific bodies, as well as by athletes who bravely shared their experiences.

Specifically, on 30 July 2025, WA announced the implementation of new regulations for competition in the female category. Noting that the regulations would be enforced by the WA starting 1 September 2025, and therefore in effect at the World Athletics Championships taking place in Tokyo from 13 to 21 September 2025, the press release clarified: “All athletes wishing to compete in the female category at the World Championships are required to undergo a once-in-a-lifetime test for the SRY gene – a reliable proxy for determining biological sex. This is to be conducted via a cheek swab or blood test, whichever is more convenient” (11). These regulations supersede the previous 2023 Eligibility Regulations for the Female Classification (Athletes with DSD), which effectively required athletes with a sex variation to take androgen-suppressing drugs to lower their testosterone levels below 2.5 nmol/L in order to compete in the female category (7). These regulations also replace the previous Eligibility Regulations for Transgender Athletes, which required trans women athletes to have transitioned before reaching 12 years of age or before puberty, whatever came first, and to maintain their testosterone levels below 2.5 nmol/L, in order to be eligible to compete in the women's competition category (26).

The new WA regulations apply to all World Rankings Competitions, which include international age group competitions as well as national and regional child age group competitions. Categories of World Rankings Competitions are listed in Table 7.5 of World Athletics Ranking Rules from 2025, ranging from the Olympic Games and World Championships to U18, Youth and National competitions (27). Given the inclusion of U18, Youth, and National competitions,^5^ this new sex testing requirement will apply to athletes under the age of 18 years^6^ who may lack legal capacity to consent to the requirement under various laws. These laws relate to the validity and enforceability of the WA regulations as a contract, consent to specimen collection and analysis as a form of medical treatment, consent to genetic testing, and consent to the processing of sensitive personal data (29–31). Regulations governing girls' and women's eligibility to compete in international sport thus raise profound ethical and legal concerns. From the IOC's initial sex verification programs to WA's more recent restrictions on women with sex variations and trans women athletes, the regulations that sports governing bodies have invoked to bifurcate women and men into two binary categories have garnered criticism for lacking scientific justification and for violating athletes’ fundamental rights (23, 32, 33). We argue that WA's 2025 decision to revive its abandoned mandatory sex testing requirement is indefensible given that the methods the organization has chosen to recycle open a barrage of legal and ethical concerns and, seemingly, ignore the problematic history of sex testing in sport.

## If that sounds familiar, it is because we've been there already!

WA's plan to use polymerase chain reaction (PCR) testing to identify portions of the SRY gene is not new. By the 1990s, the IOC and WA had abandoned the chromosomal testing that influential geneticist Albert de la Chapelle so clearly noted was inaccurate in his letter to María José Martínez-Patiño. Shifting to PCR-based genetic testing to identify the SRY gene located on the Y chromosome, the IOC launched PCR-based DNA testing prior to the 1992 Winter Olympics in Albertville, France, where women competing were screened via PCR tests for DYZ1 repeat sequences, and if positive, re-tested for the presence of the SRY gene (2, 34).

The IOC and WA sex screening requirements and policies have not always aligned. Currently, IOC President Kirsty Coventry has not yet confirmed if the IOC will allow WA to enforce its regulations at the Olympics, or if she will concede to USA President Donald Trump's demand that all women competing at the 2028 Olympics in Los Angeles undergo sex testing (35). In the meantime, Algerian boxer Imane Khelif, who was at the centre of the 2024 Paris Olympics controversy, filed an appeal with CAS on 5 August 2025 to contest World Boxing's decision that bars her from competing in events, including the 2025 World Boxing Championships, unless she undergoes genetic sex verification testing. On 1 September 2025, CAS rejected her request for a suspension of World Boxing's decision while the appeal is pending, meaning the ban remains in force for now^7^ (36). Although the IOC has not been named as a respondent in the dispute, it may nevertheless take a position that impacts the case in light of its review of sex testing practices ahead of the 2028 Los Angeles Olympic Games. The IOC's announcement on 12 September 2025, that a working group has been struck to examine how to “best protect the female category,” indicates that discussions are proceeding (37). According to the IOC, the working group will consist of experts and sports governing bodies whose identities will remain confidential “to protect the integrity of the group and their work” (37). As noted by the Sport and Rights Alliance (38, p. 1), “this opaque process stands in stark contrast to the comprehensive, transparent, and multi-stakeholder consultation that led to the development of the IOC's widely-respected [Framework on Fairness, Inclusion and Non-Discrimination on the Basis of Gender Identity and Sex Variations]”.

While the IOC and WA have, at times, mandated the same requirements, there have been points of departure as well where a consensus among leaders of both influential organizations on how to manage the women's category was not present. For example, in 1992, WA formally abandoned chromosome-based testing altogether. From then on, sex testing at international track and field competitions was based only on suspicion and assessed medically on a case-by-case basis (39). However, the IOC Medical Commission continued to conduct SRY gene analysis on 2,406 women at the 1992 Summer Olympics in Barcelona, Spain, where five athletes' tests were confirmed “positive”, which required these women to undergo additional scrutiny. Of the five, four athletes were cleared to participate in the women's events after physical exams, and one athlete withdrew (40). Four years later, at the 1996 Summer Olympic Games in Atlanta, USA, the IOC Medical Commission again screened competitors for the SRY gene, with eight of 3,387 women athletes tested returning positive test results. For these eight women, seven had partial or complete androgen insensitivity, and one woman had previously undergone gonadectomy and was presumed to have 5-alpha reductase deficiency (34). Several athletes first learned of a sex variation during Olympic testing, but at least one had been diagnosed earlier (41).

In setting eligibility rules for entry into the women's category, both sex chromatin and then PCR tests were considered improvements on the earlier practices of physical exams. Physical screening of women competitors' bodies occurred for some athletes at some international competitions in 1967 and 1968. Newspaper coverage confirms that some women were required to undergo physical examinations prior to being approved to compete. For example, prior to the 1967 Pan-American Games in Winnipeg, track and field official Doug Gudmandson told reporters from the *Winnipeg Free Press* that three doctors would be present to enforce the WA rule that all female athletes would need to verify their sex, explaining: “I hope we won't have to send any “men” home” (42, p. 56). Another Canadian newspaper reported, “Compulsory physical examinations are on tap this week for 100 Pan-American Games track and field females” (43, p. 10). While details are difficult to find, it appears that the sex testing process was restricted only to women competing in track and field events, and that women competing in other sports only required a doctor's certificate attesting they were women (44). According to one official, “the international track and field association isn't satisfied with the certificates so three female doctors will examine the girls” (43, p. 10). As American shot-put athlete Maren Seidler remembers, “They lined us up outside a room where there were three doctors sitting in a row behind desks. You had to go in and pull up your shirt and push down your pants. Then they just looked while you waited for them to confer and decide if you were O.K… It really was hideous … I just felt that it was humiliating” (45, p. 9). Members of the IOC Medical Commission soon noted that they preferred the more clinical and less invasive approach of chromosome analysis (46). Once made aware of the embarrassment the physical exams created, the IOC moved to find better and less objectionable methods of sex screening to use at the 1968 Olympic Summer and Winter Games. The first option pursued was sex chromatin (47).

As a simple and painless test that could be conducted by scraping the inside of the cheek with a small spatula, sex chromatin testing eliminated the assault on dignity that physical examinations for sports eligibility purposes produced. An athlete's cheek cell sample could be viewed under a microscope to determine if a competitor's karyotype was 46,XX or 46,XY. However, despite the promise of using clinical methods to determine sex, as outlined by Dr. de la Chapelle above, the test was insensitive, inaccurate, and could not produce the results that the IOC sought. With this test, numerous athletes were told that their results confirmed they were not female and thus that they were ineligible to compete (40).

To improve the accuracy of tests, PCR testing replaced the sex chromatin tests to more effectively detect the SRY gene in athletes' samples. As Dickinson and colleagues (40) note, “The shift to PCR-based techniques replaced one diagnostic genetic test with another but did not alleviate the problems” (p. 1541). Attempts to simplify the complex biology of sex into one of its elements were bound to fail because sex is a multidimensional biological construct shaped by the interaction of chromosomal, gonadal, hormonal, anatomical, and secondary characteristics, as well as neuroendocrine patterns. While these elements often align to produce what is typically categorized as male or female, natural variation in individuals means that they do not always coincide in a straightforward way.

In sport, regulatory approaches that rely on testing a single marker of sex (such as chromosomes, hormone levels, or external genitalia) are therefore bound to fail, because no single biological element can capture the full complexity of sex. The absence of the SRY gene usually indicates an XX individual, but in rare cases, an individual can develop male characteristics without the presence of the SRY gene, due to alternative genetic pathways or translocation of the SRY gene to another chromosome (48). In addition, the presence of the SRY gene typically indicates an XY karyotype, but not always a male phenotype. This is the case for individuals with Complete Androgen Insensitivity Syndrome (CAIS) like María José Martínez Patiño who, despite having XY chromosomes and an SRY gene, do not respond to testosterone, and therefore develop female bodies.

In June 1999, responding to mounting criticism from medical experts, athletes, and the IOC Athletes' Commission, the IOC ended compulsory PCR sex testing for the SRY gene. Starting with the 2000 Sydney Olympics, widespread, mandatory sex verification was replaced with suspicion-based testing only, which remained in effect until 1 May 2011, when the WA's Hyperandrogenism Regulations were introduced (2). For these particular regulations, it was clear that perception was the trigger for testing (see Table 1). Reflecting on the end of the first era of sex testing, Genel and Ljungqvist (34) addressed in an essay published in the *Lancet* in 2005 the rationale for the cessation: “With the increasing number female participants, the cost of administering the test, including confirmation and counselling of detected individuals, was substantial with limited return” (p. 366). Moreover, they concluded, “laboratory-based genetic screening for female gender in sport is history, saving a lot of embarrassment - and money” (34, p. 366). Unfortunately, this is not the case, as a second era of systemic sex testing has begun in track and field, and possibly in all sport starting from the Olympics in Los Angeles in 2028 (35).

**Table 1:** The hirsutism scoring sheet that was included in WA's 2011 Hyperandrogenism Regulations.^8^

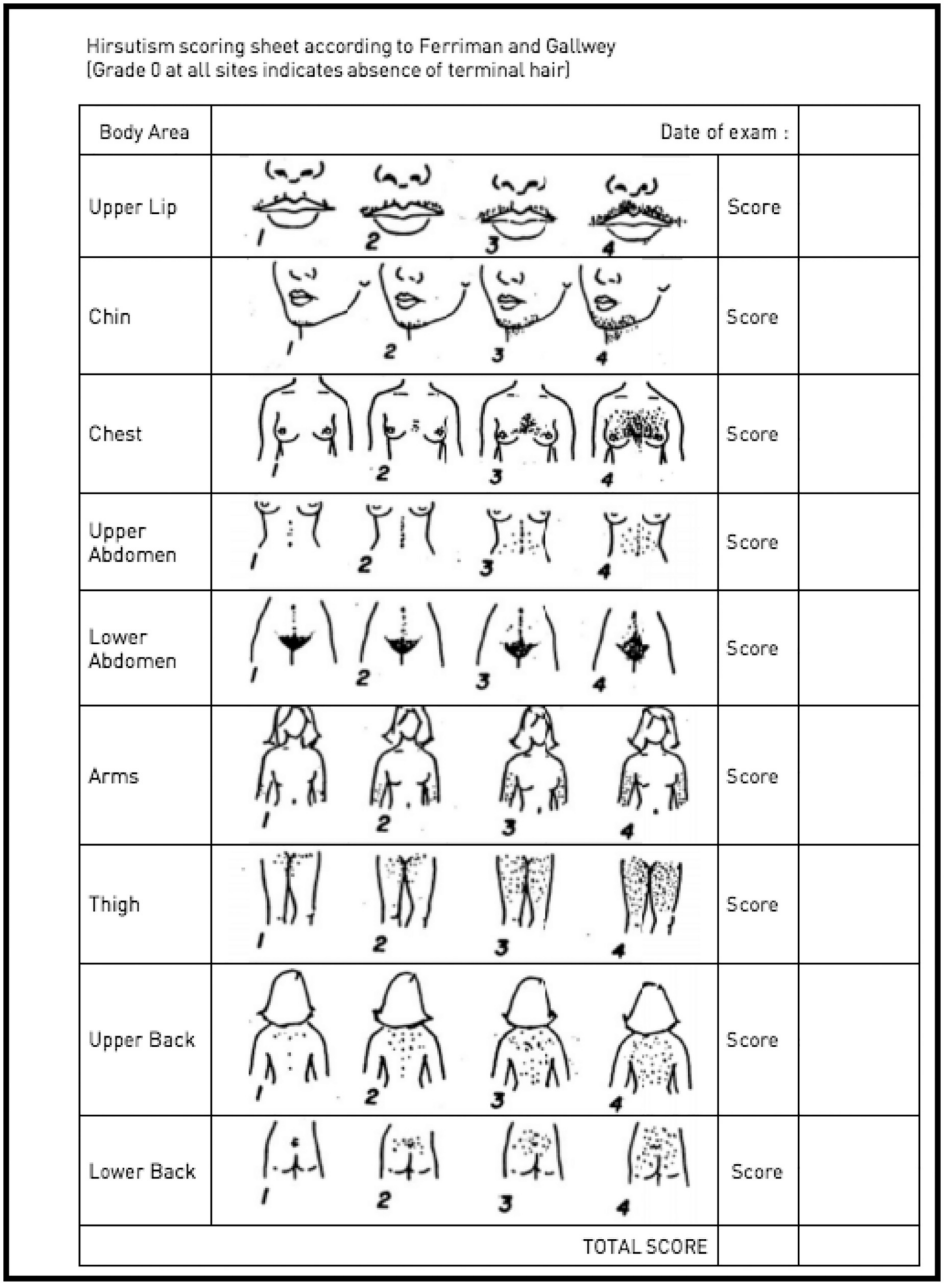

## The return of systemic sex testing in track and field

WA's confirmation in July 2025 that genetic sex testing would again be mandatory for participation at international track and field events contradicts its decision in the 1990s to abandon the discredited tests. What has changed in the near three decades since WA chose to stop requiring women undergo sex testing to compete in sport, years before the IOC made the same decision? How did we go from there, to here? Mirroring, in a way, the hesitations raised by Murray Barr regarding the implementation of the Barr Body test for sex testing purposes in sport, the discoverer of the SRY gene, Andrew Sinclair (49), came forward following the WA's announcement of the new regulations, to remind the public that the SRY gene is not a foolproof method of ensuring fairness in sport (3). Specifically, he cautions: “World Athletics asserts the SRY gene is a reliable proxy for determining biological sex. But biological sex is much more complex, with chromosomal, gonadal (testis/ovary), hormonal and secondary sex characteristics all playing a role. Using SRY to establish biological sex is wrong because all it tells you is whether or not the gene is present. It does not tell you how SRY is functioning, whether a testis has formed, whether testosterone is produced and, if so, whether it can be used by the body” (3). While this is acknowledged in the WA's “new” regulations as an exception, to the best of our knowledge, these individuals are referred now for the first time to as “biological males who have [CAIS]” (12). WA's Eligibility Rule 3.5.1 states that ““biological male” means someone with a Y chromosome and “biological female” means someone with no Y chromosome.” Moreover, WA clarifies in Rule 3.5.2 that people with a sex variation (including CAIS) are “biological males” (12, p. 2).

WA's updated regulations require that athletes carrying the SRY gene take on the responsibility of convincing the WA Medical Manager that they have CAIS, in order to be eligible to compete in the female category. The Regulations for the Implementation of Eligibility Rule 3.5 specify that “it will be for the Athlete to demonstrate to the satisfaction of the Medical Manager that the Athlete has [CAIS] and therefore is eligible to compete in the female category at World Rankings Competitions” (13). Yet, the regulations provide no direction on what type of evidence would fulfill this requirement or how the Medical Manager will decide that the obligation has been met (50). The regulations only state that “the Athlete must provide a comprehensive medical history to the Medical Manager” and that the “Medical Manager may make such enquiries or investigations as they consider necessary to determine accurately and effectively whether or not the Athlete has [CAIS], including requesting further information from the Athlete and/or the Athlete's physician and/or obtaining additional expert opinion(s)” (13, p. 5). What form of proof about an athlete's biological condition would be acceptable is not made clear, particularly for athletes with Partial Androgen Insensitivity Syndrome (PAIS). Impassioned appeals to geneticists, like Martínez Patiño's to Dr. de la Chapelle, may again become necessary.

As medical geneticist and Director, Institute for Clinical and Translational Science, University of California, Irvine, Professor Eric Vilain^8^,^9^ notes, “A critical aspect of patient-centered care is diagnostic accuracy” (51, 13). Furthermore, “many individuals receive a working diagnosis of [PAIS], whereby an impaired tissue response to testosterone during development results in atypical external genitalia in 46,XY individuals. However, in only a minority of suspected *de novo* PAIS cases is a mutation actually found in the Androgen Receptor (AR) gene106,143,144. Those misdiagnosed may carry mutations in SRD5A2, NR5A1/SF-1, or LHCGR that can partially mimic PAIS” (51, 14). Identifying a variant is thus only the initial step of an investigation, not a conclusive decision about an athlete's eligibility.^10^ It is not clear from the regulations provided that WA has even thought about this, let alone worked out the logistics of establishing guidelines surrounding variant interpretation of androgen insensitivity by trained medical geneticists.

Hence, the regulations as written are inadequate as they fail to address that additional evaluation should involve medical specialists, such as gynecologists or physicians, who can examine physical traits including clitoromegaly, external genital symmetry, degree of breast development, distribution of pubic hair, and genital palpation (52). The regulations also do not specify what standard of proof the Medical Manager will apply to determine whether an athlete has met their burden of proving that they have CAIS. For example, is the standard of proof a balance of probabilities (the standard in civil cases), a comfortable satisfaction (the standard in anti-doping and sport disciplinary cases), or beyond a reasonable doubt (the standard in criminal law cases), or some other standard?

The lawfulness of assigning the burden of proof to athletes in these circumstances is questionable, and mirrors past concerns brought to CAS regarding who should have the burden to prove that an advantage is unfair. In Blake Leeper v. IAAF (53, paragraphs 279, 339–359), the CAS panel ruled that, in prohibiting the use of a mechanical aid (specifically a prosthetic limb) outside of Paralympic or disability sport events, WA's regulations constituted an unlawful form of discrimination that violated WA's own Constitution as they placed the burden on the athlete to demonstrate that their mechanical aid did not provide a competitive advantage over able-bodied athletes. The panel found that placing the burden of proof on the athlete to demonstrate a lack of advantage was not necessary, reasonable or proportionate, and was therefore invalid and unlawful (53, paragraphs 343 and 359).

In reaching the above conclusion and finding that the burden of proof should be placed on WA, instead of the athlete, the CAS panel considered several key factors, many of which are applicable to WA's new sex testing regulations. First, the panel was not persuaded by the argument that the athlete was in the best position to gather the evidence to satisfy the burden due to being in control of such evidence (53, paragraph 342). The panel noted that, if an athlete refused to undergo testing or provide relevant information to WA without reasonable justification, then that refusal could be considered when determining whether WA had satisfied its burden to prove an unfair advantage. Similar reasoning applies to the WA's new sex testing regulations, as it is the athlete who will control access to the sensitive personal data needed to prove the existence of CAIS. Second, the panel found that satisfying the burden would involve complex factual and scientific enquiries likely to be challenging, expensive, and time-consuming for an athlete due to the need to retain experts without the assistance or financial support of WA (53, paragraphs 344–349). As noted above, assessing androgen insensitivity is a complex factual and scientific matter that requires the expertise of medical specialists to evaluate degrees of virilization. While the regulations state that, at the request of an athlete, WA will “propose and appoint an independent advisor” to help the athlete understand the assessment process, “offer professional counselling or such other support services *as the Medical Manager deems appropriate in the circumstances*” (emphasis added), and pay the costs of such advisory, counselling and support services, it remains to be seen whether this will provide adequate support for an athlete attempting to satisfy their evidentiary burden. This skepticism is justified in light of the significant discretion given to the Medical Manager to offer and therefore fund support services, and WA's history of retaining experts with conflicts of interest (see Camporesi et al., forthcoming). Third, the panel in the Leeper case found that WA's rules for mechanical aids did not provide any clear, accessible and structured process with timelines that must be followed to determine whether an athlete met their burden of proof (53, paragraphs 350–354). As explained above, the same can be said of WA's sex testing regulations as they do not sufficiently describe what evidence is needed to confirm the existence of CAIS (aside from the provision of an “comprehensive medical history”), how such evidence should be submitted, how that evidence will be evaluated, and whether an athlete can challenge the Medical Manager's reliance on “additional expert opinion(s)” that might conflict with the athlete's evidence (13, p. 5). The absence of timelines for considering an athlete's evidence is also concerning because it can lead to a significant delay and extend the period of time during which the athlete is ineligible for competition, as noted by the CAS panel in the context of Leeper's case (53, paragraph 354). Fourth, and finally, the CAS panel in the Leeper case found that placing the onus on an athlete to prove the absence of a competitive advantage was neither necessary nor proportionate as it led to any doubt about the existence of such an advantage to be resolved *against* the athlete by denying them eligibility (53, paragraph 356). Again, similar reasoning can be applied to WA's sex testing regulations as the athlete has the burden of proving an absolute – that is, *complete* insensitivity to androgens. Any doubt regarding the athlete's sensitivity to androgens based on a subjective evaluation of their physical traits would seem to be resolved against the athlete, and result in their ineligibility from competition.

Genetic tests for the SRY gene, while less intrusive than prior methods, continue to raise ethical questions about why only women must prove their sex. For example, prior to the 1976 Winter Games in Innsbruck, reporter Will Grimsley raised the issue of the continued use of cheek swabs to verify sex. Quoting some of the 248 women required to participate in the cheek swab verification process in Innsbruck, Grimsley highlighted several athlete voices, including a luge athlete who confided, “I resent it – it is a challenge to our identity,” and another athlete who asked, “If the women must take sex tests, why not the men? I've seen some male figure skaters that I think might well be included – but for heaven's sake, don't use my name” (54, p. 24). Despite these ethical concerns, WA's return to required SRY genetic testing only applies to the female competition category, not the male competition category (12, at rule 3.5.4).

Highlighting several concerns with the reintroduction of genetic sex testing in sport, the European Society for Human Genetics has put forward a statement urging caution regarding these tests. While the statement does not explicitly condemn the introduction of the testing and appears cautious in its stance, given the multifaceted ethical and legal challenges raised by the policies, the position of the organization is that the “result of such a test cannot determine completely whether an individual should compete in the female category. Fine-tuning of protocols and exceptions will take time and careful consideration. It will also be very important that athletes receive medical and psychosocial support in the case of an unexpected test result” (55).

WA's new regulations are also likely to be unlawful under anti-discrimination, data protection, and genetic testing laws. The right to non-discrimination is enshrined in international, supranational and national laws [e.g., (8, 56, 57)], as well as WA's own Constitution (58) and the IOC's Olympic Charter (59). The new regulations are *prima facie* discriminatory as they make distinctions on the basis of sex, gender identity, and genetic characteristics. As a result, WA will need to defend the discrimination as being necessary, reasonable, and proportionate (9); however, this will be difficult to do in light of the lack of scientific evidence and consensus about the reliability of the SRY gene test and whether an athlete with the SRY gene who does not have CAIS has an unfair competitive advantage over other women athletes. The lack of such scientific evidence and consensus in relation to trans women athletes was recently relied upon by a Brussels court to declare that eligibility regulations adopted by Union Cycliste Internationale (the international federation for cycling) were discriminatory and therefore null and void (60, 61).

Data protection laws apply to WA's processing (i.e., collection, storage, and use) of athletes' genetic and health data under the new regulations. These data protection laws often require an organization to have a valid legal ground to process such sensitive personal data – specifically, the explicit consent of the data subject or some other legal basis laid down in law (62).^11^ The WA's new regulations purport to rely on the consent of athletes to process their sensitive personal data. The regulations state that an athlete who wishes to be eligible to compete must consent to the processing of their sensitive personal data for the purpose of implementing the regulations (13, p. 5). Such consent is likely to be invalid under data protection laws as it is not voluntary, informed, or explicit (62). The consent is coerced, and not voluntary, as athletes have no choice but to agree to the data processing if they want to continue participating in their sport. This threat of exclusion from athletics due to the withholding or withdrawing of consent to data processing is expressly acknowledged by WA in the regulations (13, p. 6). The consent is not informed because athletes do not have access to appropriate genetic counselling to understand the potential consequences of receiving genetic test results prior to consenting (50). Access to such counselling is only contemplated in the regulations *after* the athlete has undergone the genetic test and is found to have the SRY gene (13, p. 6). Lastly, the consent of athletes will not be explicit in all cases, as the regulations indicate that, where an athlete does not provide written consent to the processing of their personal data, their consent will be implied by virtue of their participation in athletics (13, p. 5). If the consent of athletes is not valid, then WA's processing of the sensitive personal data will be unlawful, which will allow athletes to exercise their rights to administrative and judicial remedies under data protection laws to stop the data processing, obtain financial compensation for the harms they have suffered from the non-compliant data processing, and require WA to change its policy approach to regulating eligibility (62).

Finally, with respect to national genetic testing laws [e.g., (63–65)], Erikainen et al. (31) note that WA's regulations violate several key principles, including (1) the prohibition on genetic testing for non-health purposes; (2) the prohibition on using genetic testing as a condition for receiving benefits or other discriminatory purposes; and (3) the prohibition on genetic testing without free and informed consent. The existence of such national genetic testing laws will make the implementation of sex testing in some countries extremely challenging, as it appears that some athletes will be required to undergo genetic testing in a nation other than their own. For example, prior to the 2025 World Championships in September 2025, French athletes learned that they would be unable to obtain the SRY gene test in France as it was prohibited under domestic bioethics legislation, and that they would thus need to obtain the test abroad (66).

The logistics of implementing WA's July 2025 announcement prior to 1 September 2025 for all women seeking to compete at the 2025 World Championships taking place in Tokyo, Japan, between 13 and 21 September 2025 is also noteworthy. The rushed implementation of the requirement has caused operational challenges in ensuring the necessary tests can be completed in the short timeframe provided. For example, Canada's rollout of tests for its women track and field athletes at the Canadian national championships was unsuccessful when the lab contracted to carry out the necessary tests for the Canadian women realized the test kits used did not meet WA's requirements. The error left the entire Canadian delegation without the necessary test results less than a month before the due date, without a plan in place to acquire the correct test kits (67).

As it stands, WA is requiring the use of old tests on a new generation of athletes in a new legal context that provides greater protections of athletes' rights than past eras under anti-discrimination, data protection and genetic testing laws. Sinclair bluntly acknowledges, “I, along with many other experts, persuaded the International Olympic Committee to drop the use of SRY for sex testing for the 2000 Sydney Olympics. It is therefore very surprising that, 25 years later, there is a misguided effort to bring this test back. Given all the problems outlined above, the SRY gene should not be used to exclude women athletes from competition” (3). Contrary to Sinclair, we argue that it is not, unfortunately, very surprising that this test is being brought back given WA's resistance to public scrutiny and legal accountability, as well as the trajectory of its policy in the last decade from one of dissuasion to one of exclusion for athletes with sex variations.

## Unpacking WA's rationales for reviving systemic sex testing of women athletes

WA's new sex testing regulations are the result of many years of policing by WA of female bodies. From 2011 to 2025, there have been three subsequent iterations of the regulations requiring women with sex variations to lower their endogenous testosterone levels from 10 to 5 to 2.5 nmol/L. This progressive lowering of the limit served as a strategy of dissuasion from participation for women with sex variations.^12^ Athletes were offered the “opportunity” to participate if they agreed to medicalize their bodies, resulting in serious physical and psychological harms as courageously explained by Dutee Chand, Evangeline Makena Kathenya, Annet Negesa and Margaret Wambui in the documentary film, Category: Woman.

One might wonder why WA no longer considers its prior strategy of dissuasion from participation via regulations setting upper thresholds for testosterone to be sufficient. One possibility is that the regulations caused too many problems, as evidenced by the direct and indirect challenges to the regulations filed with CAS (8, 9), the Swiss Federal Tribunal (68), the European Court of Human Rights (19), and a data protection regulator (69). Ironically, however, the new regulations are likely to give rise to a wave of new legal challenges that may be more difficult for WA to defend than the previous regulations (33, 70).

The new regulations function to move, yet again, the goalposts of what is required for women athletes with sex variations to compete without adequate scientific evidence. Recall that in response to the suspension of the 2011 Hyperandrogenism Regulations by CAS, WA replaced the suspended regulations with regulations that applied only to specific events – the events at which Caster Semenya excelled – and that lowered the allowable threshold for endogenous testosterone from 10 to 5 nmol/L in 2018. As concluded by Pielke and co-authors in their independent critique of the scientific research submitted by WA in support of the 2018 regulations, “We find this research to be deeply flawed and uncorrected even after the errors were called to the attention of [WA] and the scientific journal which published them” (71, p. 2). Moreover, Franklin and colleagues (72) concluded the statistical evidence that the data produced by WA in favour of lowering the testosterone limit from 10 to 5 nmol/L was “more likely to have arisen by chance” (p. 1). In other words, WA had cherry-picked a few events (the middle-distance ones in which Semenya was competing) and applied restrictions on athletes only for those events. This constitutes a seriously wrong application of scientific findings (73). However, we argue, these breaches of scientific integrity were given insufficient weight by the CAS panel in Semenya's case [see (9), paragraphs 517–538, and 573].

Subsequently, in 2023, WA revised the regulations to apply to *all* track and field events at World Ranking Competitions, reduce the allowable testosterone threshold to 2.5 nmol/L, and extend the period of time in which an athlete must maintain their testosterone level below this threshold from 6 months to 24 months. Again, this was done without any publicly available peer-reviewed scientific evidence, something which was directly legitimized by CAS in their 2019 award. In this light, the decision to reintroduce sex testing for all women athletes in 2025 is not surprising, as it is an act of moving the goalposts once again (50).^13^

There are several ways in which WA may attempt to insulate its regulatory actions from public scrutiny and legal accountability. One strategy may be to secure favourable decisions from CAS about its autonomy to make decisions for its sport. This may involve asking a CAS panel to narrow the type of expert evidence relied upon in an arbitration proceeding challenging the regulations, including disregarding expert witness claims concerning the degree of advantage conferred by other biological or genetic variations (17), or disregarding expert witness claims grounded in human rights' concerns, as was seen in Semenya's case [see (9), paragraphs 554, 555, 587 and 588]. Moreover, the lowering of the bar for the admissibility of scientific evidence to be set at that of a “reasonable person in good faith” negates expert testimony by human rights experts (74, 75). While the decision of the European Court of Human Rights in the Semenya case regarding the right to a fair hearing may help mitigate these concerns (19), questions remain about the independence and impartiality of CAS (70). However, this highlights the importance of challenging the regulations on multiple fronts, such as pursuing administrative and judicial remedies under data protection laws, in addition to bringing non-discrimination claims to CAS (33).

That said, WA's new regulations also reveal an attempt to reduce the risk of violating data protection laws. Under the predecessor 2023 regulations, WA asserted authority to monitor athletes’ compliance with blood testosterone thresholds by using doping data (7), such as the doping data stored in the World Anti-Doping Agency (WADA)'s Anti-Doping Administration and Management System located in Montreal, Canada. However, this use of doping data for sex testing purposes has been described as violating data protection laws (33) and is being investigated by Canada's Privacy Commissioner for possibly violating Canadian privacy laws (69). WA's new regulations appear to sidestep this legal risk by replacing the monitoring of testosterone levels with the SRY gene test. To the extent that the monitoring of testosterone levels remains necessary under the new regulations (e.g., for an athlete with the SRY gene attempting to prove that they have CAIS), the athlete is responsible for disclosing such data to WA, upon request of the Medical Manager (13). By placing the onus on the athlete to disclose the testosterone-level data to the Medical Manager, WA is attempting to ensure that the disclosure occurs with the explicit consent of the athlete, as required under data protection laws. However, as noted earlier, the athlete's consent is a fiction due to coercive circumstances in which it is granted.

Finally, WA's rushed rollout of the new regulations combined with its decision to delegate responsibility for ensuring compliance with the genetic test requirement to its national federation members prior to the 2025 World Championships is arguably an attempt to shield WA from liability under the many national laws that may prohibit the test. It is unlikely that there was sufficient time for any athlete to commence a legal challenge to the genetic testing requirement in their country,^14^ and for the athletes whose national laws prohibited the genetic test (e.g., France) the quickest solution was to have the test conducted in another country without such laws.

All of these actions by WA contribute to an unease about the continued self-regulation of international sport. In moving the goalposts and changing the regulations once again, without new peer-reviewed scientific knowledge to justify such changes, WA attempts to escape the gaze of public scrutiny and legal accountability.

## Conclusion

In this paper we have argued that WA's 2025 decision to revive its regulations that were abandoned in the 1990s, requiring all elite women athletes to undergo SRY gene analysis, and bringing back a new era of mandatory genetic sex testing contravenes numerous laws, and recycles old models that were abandoned for good reasons. In doing so, this paper contends that the return of systemic sex testing of women to compete in sport is indefensible on scientific, legal and ethical grounds.

These scientific, legal and ethical objections to sex testing are powerfully conveyed and encapsulated by the experience of María José Martínez Patiño and her correspondence with renowned geneticist Dr. Albert de la Chapelle. Sadly, the harms caused to Martínez Patiño's dignity, privacy, athletic career and personal relationships are not isolated and have been experienced in varying degrees by numerous women athletes who were subjected to genetic sex testing in past decades. Unfortunately, this dark history is repeating itself due to WA's 2025 decision to reintroduce this anachronistic practice, which will profoundly affect the lives and athletic careers of many more women athletes, despite warnings from medical experts (including those who developed the genetic test) and cogent arguments that genetic sex testing is unethical and violates human rights laws. We hope this paper adds to the discourse on the topic to inform perspectives, including those of WA, other sports governing bodies that have or are contemplating similar regulations, as well as athletes and their supporters who seek to challenge sex testing in sport through activism and legal advocacy.

## Footnotes

^2^World Athletics (WA) was known as the International Amateur Athletic Federation and then the International Association of Athletics Federations, or IAAF, until rebranding in 2019. For simplicity, we use the acronym WA throughout this paper to refer to the international governing body of track and field, rather than switch back and forth between IAAF and WA when referring to the periods before and after the name change.

^3^Letter to Dr. Albert de la Chapelle from María José Martínez Patiño, 25 March 1988. Permission provided by María José Martínez Patiño on 25 September 2025.

^4^Letter to Miss María Martínez from Albert de la Chapelle, M.D., 8 March 1988. Permission provided by Henrik de la Chapelle as executor of Dr. Albert de la Chapelle's estate on 17 September 2025.

^5^A list of some of the lowest scoring World Rankings Competitions to date in 2025, including child and youth age category competitions, can be accessed here: https://worldathletics.org/records/competition-performance-rankings?year=2025&sortBy=score&page=13

^6^Historically, very young athletes have competed at the highest levels, with a recent example including 15-year-old Kelly Doualla winning the 100-metre at the European Athletics U20 Championships in August 2025 in Tampere, Finland (28).

^7^A hearing has yet to be scheduled at the time of proofreading this article (14 January, 2026).

^8^This scoring sheet has since been removed from WA's website following the suspension of the regulation by a CAS panel on 24 July 2015, but a copy can be accessed here: https://silviacamporesiresearch.org/

^9^Professor Eric Vilain has served as an adviser/consultant to the International Olympic Committee (IOC) Medical Commission from about 2011 and was consulted in drafting and revising policies on athletes with sex variations in 2018.

^10^The authors thank Dr. Vilain for raising this point.

^11^See, for example, the European Union's General Data Protection Regulation, as discussed in (62).

^12^This argument is the focus of Camporesi & Teetzel, The Oxford Handbook Sport Ethics, edited 2026 in press. https://forskning.ruc.dk/en/publications/the-oxford-handbook-of-sports-ethics

^13^An argument can be made that the goalposts have remained the same for women athletes with sex variations who were complying with the previous Eligibility Regulations for the Female Classification (Athletes with DSD) (7), as the transitional rules in the new regulations allow these athletes to remain eligible for the female competition category if they continuously maintain their serum testosterone level below 2.5 nmol/L (13). However, for all other women athletes with sex variations, it is fair to say that the goalposts have moved.

^14^For example, in August 2025, a complaint against WA was filed with Japan's Personal Information Protection Commission. The complaint alleged that that WA's imminent processing of sensitive personal data (e.g., genetic data) at the upcoming 2025 World Championships in Tokyo from 13-21 September 2025 would violate Japan's Act on the Protection of Personal Information and that urgent action was required. The Commission acknowledged receipt of the complaint on 1 October 2025, and therefore it appears that there was insufficient time to investigate the complaint prior to the World Championships.
