# Supplementary material for: Sex testing in women's sport: historical harms, contemporary risks, and World Athletics' 2025 policy shift
Source: Front Sports Act Living. 2026 Feb 10;7:1723127. doi: 10.3389/fspor.2025.1723127 (PMC12930467; doi:10.3389/fspor.2025.1723127)
Supplement: Supplementary file 1 [file Supplementaryfile1.pdf]

María Martínez
[redacted]
28010 MADRID Spain

Dr. Albert de la Chapelle
Department of Medical Genetics
University of Helsinki
00290 Helsinki
FINLAND

Madrid, 25th of March, 1988

Dear Sir:

I am a spanish girl of 24 years old. I began to practice Athletics from the age of thirteen. My family lives in La Coruña (North of Spain), and I came to Madrid so that I could practice athletics in better conditions tracks with the spanish team junior; I was 17 th years old.

In 1983, I took part in World Championship in Atletics in Helsinki. My perfomances:

100 mts. Hurdles:	13.71	
100 mts.	: 11.97	
200 mts.	: 24.29	
Long Jump	: 5,91	mts.

As you can see my perfomances are find. In this Championship, I did the sex-control in Helsinki, and I did not have any problem there. I took part in the European Cup in Portugal (13.88- 100 m.h.). I had my female certificate.

In 1984 the only brothers I had, died with 15 years of age. He had leukemia, = and I gave him part of my osseus medulla for two transplant. This experience has been very hard for me, and because of that, (Ihad a strong anemia),- I could not go to the Olimpic Games in Los Angeles.

In 1986, I went to Kobe (Japan), to compete in the University Games . I did not carry my certificate of IAAF. The japaneses did the sex - control againg. They discover a cromosomic alteration:

[redacted]

" Karyotype analisis by Q-banding method revealed her sex chromosome constitution was XY." Was done from the peripheral blood.

When I came back to Spain a doctOR, member of the Spanish expedition, told a journalist. The journalist ~~siu~~ published the event in newspapers and magazines, T.V. etc. And as a result of that, every body knew about it. My federation and not support me, and they speled me from the residence sport (the place where I lived). I left the University and so I had to work because I had no money to live. I have lost my friends, and my boy-friend left me when the event came to light; Twelve years of hard work for nothing.

I thought I was going to die. I lost my licence and my perfomances. It looked as if I were a man instead of a woman.

Little by little I had recovere myself and I have received some treatment from the best specialist in Medicine.

Endocrinology and Intern Medicine (Hormone)
Gynecology (Laparoscopy, Examination, Ecography)
Genetics

Diagnostic: MORRIS' SYNDROME or TESTICULAR FEMINIZATION

Female body proportions: Normal

I understand it is very difficult to obtain a modification of reglamentation of Internacional Amateur Atletic Federation, but I think, this is not imposible consider special cases. I hope my sacrifice will not be left in oblivion. While I live I´ll go on fighting. But I am alone. I need your help.

I need you support, I Know deeply your works based on medical research about the subject in Cell, Jama, Lancet.

I have sent a letter to Mr. Samaraoch and I have the acknowledgment of the King of Spain.

I would like that this case were known by specialist in Genetics and sports Medicine.

The doctors have given me the impulse to continue fighting, and they have send that my descalification is not Fair; the results confirm: I am really a woman, even so my federation not let me participate in competitions with woman.

Please, sir, I need your help. If you wish I should try to travel to Helsinki or to another part where you were.

I wonder what are my possibilities and if it is possible, before I die, to compete againg. I will go on fignting and I hope to find someone in the world that will help me.

I send you some photographs, some medical results and a list of Doctors who know my case and support me in Spain.

I´am looking forward to receive your letter. You are my best and most important help for me. I have nothing to hide. I have not deceived anybody; and I felt ashamed and humilliated because I did not really know the subject in itself.

Please, sorry for this long letter, but it was necessary, so that you would know me.

I have lost everything and I need your help.

Yours faithfully.

María José Martínez Patiño

UNIVERSITY OF HELSINKI

DEPARTMENT OF MEDICAL GENETICS

HAARTMANINKATU 3
00290 HELSINKI
FINLAND
TEL. 358-0-418 511

March 8, 1988

Miss Maria Martinez
[redacted]
28010 Madrid
Spain

Dear Miss Martinez:

The documents you sent me, including medical reports, results of a chromosome investigation, and clinical and laboratory examinations convincingly show that you have a condition called testicular feminization syndrome. The syndrome is caused by a mutation in a gene carried by the X chromosome. The karyotype is 46,XY.

The most typical clinical features of the testicular feminization syndrome include: normal female external genitalia, normal female body proportions and muscle strength, and an entirely normal female psychosexual orientation. In other words, except for the abnormal internal genitalia caused by a metabolic abnormality, individuals with this syndrome are females. The pictures you sent me of yourself also indeed clearly show that you are a female.

You should certainly be allowed to compete with other women in sports. Excluding you from athletic activities is an unfortunate and inexcusable mistake that, in my opinion, should be corrected.

The rules of the International Olympic Committee, the International Amateur Athletics Federation, and other sports organizations requires that women should pass a screening test of X-chromatin and Y-chromatin to "prove" their "femininity." In your case, both the X and the Y chromatin will show an "abnormal" result. However, the rules of the sports organizations also explicitly state that when the chromatin tests are "abnormal," further investigations, including a chromosome study and clinical investigation by a gynecologist, should be carried out before any decisions are made.

If your case had been correctly handled, you should have undergone these examinations in Japan, and as a result, you should have been allowed to compete, and a "femininity card" should have been issued to you. Your case and those of many, many others is a tragic illustration of the inadequacy of the sex chromatin screening procedure. I and many other scientists have for many years tried to convince the sports organizations to abandon the sex chromatin screening so that further mistakes of this type can be avoided.

Miss Martinez
March 8, 1988
Page two

Unfortunately, the sports organizations, notably the IOC and the IAAF, have not yet decided to abandon the sex chromatin screening. They have refused to do so even after they have come under severe pressure from scientific organizations, which have passed resolutions asking them to reconsider their procedures in order to avoid further human suffering. Among the scientific societies that have passed such resolutions in 1987 and 1988 are:

-The Lawson Wilkins Pediatric Endocrine Society
-The American Academy of Pediatrics
-The American Society of Human Genetics
-The Australasian Society of Human Genetics
-The Canadian College of Medical Geneticists
-The American College of Obstetricians and Gynecologists
-The Endocrine Society
-The American College of Physicians

In my opinion, your case should be handled independently of the decision that I hope the sports organizations will make about sex chromatin screening. As indicated earlier, it is in full agreement with the policies of the International Olympic Committee that you are given a "femininity card" and allowed to compete with other women. I can only hope that the responsible organizations will handle your case in this way.

Yours sincerely,

Albert de la Chapelle, M.D.
Professor and Chairman

AdlC/bo
B01
